# Robot-assisted and conventional therapies produce distinct rehabilitative trends in stroke survivors

**DOI:** 10.1186/s12984-016-0199-5

**Published:** 2016-10-11

**Authors:** Francisco J. Valero-Cuevas, Verena Klamroth-Marganska, Carolee J. Winstein, Robert Riener

**Affiliations:** 1 Department of Biomedical Engineering, University of Southern California, 3710 McClintock Ave, RTH 404, Los Angeles, CA 90089-2905 USA; 2 Division of Biokinesiology and Physical Therapy, University of Southern California, Los Angeles, CA USA; 3 ETH Zurich and University of Zurich, Zurich, Switzerland

## Abstract

**Background:**

Comparing the efficacy of alternative therapeutic strategies for the rehabilitation of motor function in chronically impaired individuals is often inconclusive. For example, a recent randomized clinical trial (RCT) compared robot-assisted vs. conventional therapy in 77 patients who had had chronic motor impairment after a cerebrovascular accident. While patients assigned to robotic therapy had greater improvements in the primary outcome measure (change in score on the upper extremity section of the Fugl-Meyer assessment), the absolute difference between therapies was small, which left the clinical relevance in question.

**Methods:**

Here we revisit that study to test whether the multidimensional rehabilitative response of these patients can better distinguish between treatment outcomes. We used principal components analysis to find the correlation of changes across seven outcome measures between the start and end of 8 weeks of therapy. Permutation tests verified the robustness of the principal components found.

**Results:**

Each therapy in fact produces different rehabilitative trends of recovery across the clinical, functional, and quality of life domains. A rehabilitative trend is a principal component that quantifies the correlations among changes in outcomes with each therapy.

**Conclusions:**

These findings challenge the traditional emphasis of RCTs on using a single primary outcome measure to compare rehabilitative responses that are naturally multidimensional. This alternative approach to, and interpretation of, the results of RCTs may will lead to more effective therapies targeted for the multidimensional mechanisms of recovery.

**Trial registration:**

ClinicalTrials.gov number NCT00719433. Registered July 17, 2008.

**Electronic supplementary material:**

The online version of this article (doi:10.1186/s12984-016-0199-5) contains supplementary material, which is available to authorized users.

## Background

Randomized clinical trials (RCTs) of various sizes and complexity have sought to compare the efficacy of alternative therapeutic strategies for the rehabilitation of motor function in chronically impaired individuals. But differentiating the benefits of robot-assisted vs. conventional therapies for arm function in chronic stroke survivors has proven elusive because RCTs do not point to a clear clinical preference [1–5]. Physiological details and treatment protocols aside, we believe that a contributor to inconclusive results in rehabilitation RCTs could be the focus on a single primary outcome to quantify a rehabilitative response that is naturally multidimensional and longitudinal—especially because it is well known that gains in the primary outcome in chronic populations tend to be small and differ little across therapies [1–4].

As a result of this focus on a primary outcome, analyses of changes in the available secondary outcomes are considered to be only indirectly informative and speculative. In reality, however, the secondary outcomes may provide insight into why the primary outcomes changed or not. In fact, the International Classification of Functioning, Disability and Health (ICF) by the World Health Organization [6] teaches us that quantifying the multiple dimensions of body structure and function, activity and participation requires several outcomes. Seen from this perspective it is difficult to define and justify a specific selection of—and hierarchy among—primary and secondary outcomes. Thus, several rehabilitation studies have begun to explore interactions among outcomes [7–12]. Because one may intuit that different therapies may lead to different rehabilitative trends, a systematic exploration and quantitative evaluation of this idea should be performed. In addition, a prior study using the ARMin III robot in a within-subject design found that robotic therapy can elicit improvements in arm function across different outcome measures that are distinct from, and perhaps a supplement to, conventional therapy [13]. Elaborating on this multidimensional approach to rehabilitation, we now present what to our knowledge is the first example of distinct rehabilitative trends between robot-assisted vs. conventional therapies in the context of an RCT. In particular, we contrasted robot-assisted therapy as per the ARMin (an exoskeleton robot that allows task-specific training in three dimensions with assistance as needed control) vs. conventional outpatient therapy. Typical conventional outpatient therapy is a heterogeneous combination of physical and occupational therapies following various models of practice [14–16]. The goal of this study is not to reproduce the results of the prior RCT, compare across typical conventional outpatient therapies, nor suggest how to program robotic therapy differently. Rather, it is to perform a secondary analysis to test for different rehabilitative trends in that prior study.

## Methods

We tested for distinct differences between robot-assisted vs. conventional therapies in chronically impaired stroke survivors by retrospectively analysing changes in all the seven motor function outcomes from our recent prospective, multicentre, parallel-group randomized trial (ClinicalTrials.gov number NCT00719433) [2]. That RCT agreed with others by finding statistically significant, but small, changes in the primary outcome (i.e., Fugl-Meyer Assessment, score, upper motor functional part, FMA) between therapies. As in other studies of stroke rehabilitation, that left the clinical advantage of either therapy in question [1–4, 17].

Here we re-examine the data from that NCT00719433 study (Table 1) by using principal components analysis (PCA) to quantify the rehabilitative trends between the start and end of 8 weeks of therapy (Tables 2 and 3, Fig. 2 and Additional file 1). Note that the goal of this study is not to suggest how to modify the robotic and conventional therapies in that prior study. Rather, it is to perform a secondary analysis to explore rehabilitative trends in that study given that all outcome measures available to us and allow the use of PCA of all outcome measures.

**Table 1:** Outcomes included in this study, collected in RCT NCT00719433 [2]

Outcome	Link to ICF	Description
FMA	Body structure & function	Upper extremity motor function of the Fugl-Meyer Assessment [35]
WMFTf	Activity	Wolf Motor Function Test function-domain. A qualitative measure of motor performance of the affected arm in the clinical environment [27]
WMFTt	Activity	Wolf Motor Function Test time-domain. A quantitative measure of performance of the affected arm in the clinical environment [27]
Mean strength	Body structure & function	Voluntary joint torque capability as measured by ARMin. A patient’s arm is brought to predefined positions and the patient applies maximal, voluntary, and isometric torques in directions of shoulder abduction, adduction, anteversion, and retroversion, and of elbow flexion and extension. Peak torques are added to calculate the mean strength in Newton-meters. Patients in the conventional therapy group experienced ARMin only during this assessment of mean strength, but this exposure did not involve any training [2].
Grip strength	Body structure & function	As measured by a handheld dynamometer (Jamar, Sammons Preston Rolyan, Bolingbrook, IL, USA).
MAL	Activity	Motor Activity Log. A structured interview with the patient about quality of movement of the affected arm in the natural, home, and community environment [36]
SIS	Participation	Stroke Impact Scale (version 2.0). A self-reported measure of health status. We used the physical dimensions score from the four domains of strength, hand function, mobility, and activities of daily living [37].

**Table 2:** Rehabilitative trends (i.e., correlations among changes in outcomes)

		Conventional		Robot-assisted	
Link to ICF	Change in outcome	1st PC trend	2nd PC trend	1st PC trend	2nd PC trend
Body structure & function	FMA	0.81	0.41	0.87	0.23
Activity	WMFTf	1	−0.64	0.76	0.83
Activity	WMFTt	0.76	−0.94	1	0.41
Body structure & function	Mean strength	0.83	0.11	−0.64	1
	Grip strength	0.59	0.13	−0.85	0.43
Activity	MAL	0.53	1	−0.18	1
Participation	SIS	0.81	0.39	−0.56	0.11
	% Contribution	31.02 %	18.48 %	30.35 %	21.16 %
	Cumulative %	49.50 %		51.51 %

**Table 3:** Details of all seven principal components

Metric	1st PC	2nd PC	3rd PC	4th PC	5th PC	6th PC	7th PC
Conventional therapy							
FMA	0.81	0.41	−0.71	−0.05	−0.10	1.00	−0.26
WMFTf	1.00	−0.64	0.20	−0.32	−0.22	0.16	1.00
WMFTt	0.76	−0.94	−0.28	−0.28	−0.21	−0.42	−0.83
Mean strength	0.83	0.11	−0.48	1.00	0.39	−0.50	0.23
Grip strength	0.59	0.13	1.00	0.63	−0.57	0.25	−0.36
MAL	0.53	1.00	−0.15	−0.47	−0.65	−0.66	0.08
SIS	0.81	0.39	0.58	−0.47	1.00	−0.05	−0.23
% Contribution	31.02 %	18.48 %	15.93 %	11.18 %	9.20 %	8.51 %	5.67 %
Cumulative %	31.02 %	49.50 %	65.43 %	76.62 %	85.82 %	94.33 %	100.00 %
Robot-assisted therapy							
FMA	0.87	0.23	0.44	−0.86	−0.60	0.44	0.58
WMFTf	0.76	0.83	0.12	0.15	1.00	0.31	0.24
WMFTt	1.00	0.41	−0.40	−0.38	−0.19	−0.50	1.00
Mean strength	−0.64	1.00	−0.26	−0.36	−0.10	−0.95	0.60
Grip strength	−0.85	0.43	−0.43	−0.79	0.09	1.00	−0.37
MAL	−0.18	1.00	0.29	1.00	−0.58	0.41	−0.30
SIS	−0.56	0.11	1.00	−0.51	0.25	−0.36	−0.60
% Contribution	30.35 %	21.16 %	15.60 %	12.97 %	8.01 %	6.66 %	5.26 %
Cumulative %	30.35 %	51.51 %	67.11 %	80.08 %	88.08 %	94.74 %	100.00 %

For each therapy we show all seven principal components (PCs) as per their loadings and percent variance explained. The 3rd to 7th PCs are included for completeness, but we refrain from interpreting them as they each explain increasingly less variance

The outcome measures obtained in that NCT00719433 study are listed and described in Table 1. Note that these are variables with different units and numerical magnitudes. Therefore, to compare them in a way that mitigates their numerical differences, we first transformed them by taking the natural log of the values [18].

**Fig. 1:**
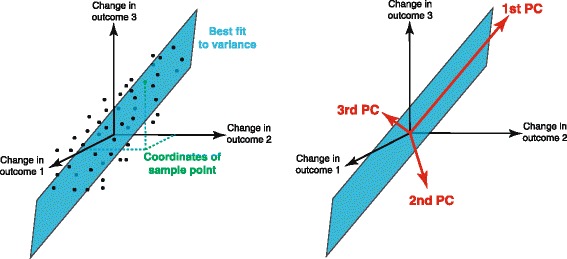
Rehabilitative trends obtained from principal components analysis (PCA). Consider the schematic case of three outcomes, where the change in each outcome with therapy is plotted for all subjects. PCA finds the best linear fit to the data using 3 perpendicular vectors: the 1st, 2nd, and 3rd principal components (PCs), labeled in descending order by variance explained. Each PC is a rehabilitative trend that quantifies the correlations among outcomes

As shown schematically for three outcomes in Fig. 1, PCA finds the best linear fit to the change in each of the outcomes between the start and end of each therapy. The visualization of a hyperplane embedded in the seven-dimensional space of seven outcomes is not possible to show graphically, but the intuition obtained in the three dimensional schematic example carries over to higher dimensions. The PCs are a set of vectors expressing the main correlations among the changes in outcome measures, where the entries in each of the vectors (known as loadings) describe the seven-dimensional changes in outcomes that best explain the effects of the RCT. Each of the seven principal components (PCs) is a column vector as shown in Tables 2 and 3.

We also tested the robustness of the rehabilitative trends found. We did so by using a permutation test [19] that randomly and repeatedly shuffled patients into two groups, A and B, of size equivalent to the conventional and robot-assisted therapies. That is, for each shuffle, all subjects were randomly assigned to one of two groups. We then repeated the PCA on each of these shuffled groups to see how likely it is for our actual results are to appear by a chance, see Additional file 1.

## Results

**Fig. 2:**
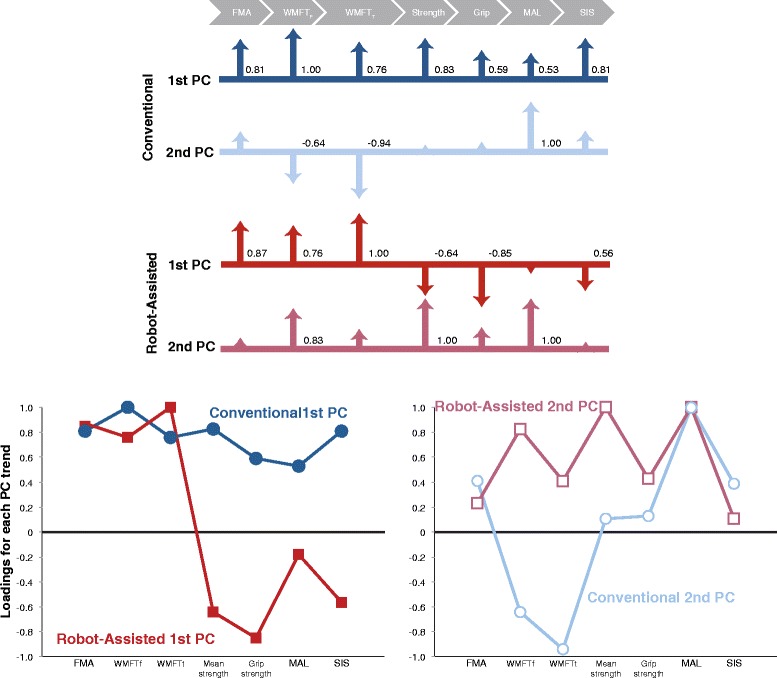
Graphical representation of the two most prominent rehabilitative trends for each therapy. The loadings of each trend quantify the correlations among changes in outcomes. *Top*: Shown as scaled arrows. *Bottom*: Shown as line plots

Table 2 and Fig. 2 describe the rehabilitative trends between the start and end of 8 weeks of therapy. These *rehabilitative trends* quantify the correlations among changes in outcomes with each therapy. They are expressed as seven principal components (PCs) in the form of column vectors^1^, Table 2. PCs are rank-ordered, with the first explaining the most change (i.e., variance), and the seventh the least. The entries of each PC, called *loadings*, specify the details of each rehabilitative trend. Tables 1 and 2 show the loadings and variance explained for all seven PCs, which are also plotted in Fig. 2. The first two PCs suffice to explain ~50 % of the variance. The 3rd to 7th PCs each explains a decreasing amount of variance in the results, ranging from 16 to 5 %. Thus interpreting them becomes increasingly uncertain. Therefore, we focus on the first two PCs and refrain from doing so and only point out that the five remaining PCs continue to show differences between therapies—which is our main finding.

In individuals receiving ***conventional therapy***, the 1st and 2nd PCs together explain 49.50 % of the total mean effect of therapy (31.02 and 18.48 %, respectively, Table 2). The details of the 1st most dominant trend (i.e., the 1st PC) show that the Wolf Motor Function Test function-domain (WMFTf, a *qualitative* measure of motor performance of the affected arm in the clinical environment) shows the biggest change, which is *positively* correlated with changes in FMA score (with a normalized strength of correlation 0.81), *and* the correlation with the other outcomes (ranging from 0.83 to 0.53). The 2nd most dominant trend (i.e., the 2nd PC), in contrast, has the Motor Activity Log (MAL, a structured interview with the patient about quality of movement of the affected arm in the natural, home, and community environment as the dominant element, with *positive* weak to moderate correlations with FMA, mean strength (a measure of voluntary joint torque capability), grip strength, and the physical domain of the Stroke Impact Scale (SIS, a combination of the four domains of strength, hand function, mobility, and activities of daily living of the complete SIS questionnaire), but strong *negative* correlations with WMFTf and the Wolf Motor Function Test time-domain (WMFTt, a *quantitative* measure of performance of the affected arm in the clinical environment).

In contrast, stroke survivors receiving ***robot-assisted therapy*** show different 1st and 2nd dominant trends explaining 51.51 % mean effect of therapy (30.35 and 21.16 %, respectively). In them, WMFTt shows the strongest effect in the 1st, most dominant trend, which is strongly and *positively* correlated with FMA and WMFTf (0.87 and 0.76, respectively). However, these are *negatively* correlated with all other outcomes. In the 2nd most dominant trend, all outcomes are positively correlated, with mean strength and MAL both sharing the most dominant effect, but weakly correlated with FMA and SIS.

In addition, permutation tests based on data shuffling allowed us to test the robustness of the details of the two main rehabilitative trends shown in Fig. 2. As described in the Additional file 1, we find that the loadings of the 1st and 2nd PCs (i.e., the two main rehabilitative trends) show distinct departures from the loadings seen in the 100 shufflings into groups A and B. That is, each of the 100 shufflings produces loadings for each PC. We then compared the distribution of all loadings values found for the random groups A and B to the actual value of the loadings found for the conventional and robot-assisted groups. As is shown in Additional file 1, we find that of the 14 possible loading values for 1st PC (i.e., 7 loading values for each of the two groups A and B), in only 4 cases did the actual value of the loadings for the conventional and robot-assisted groups overlap with the bulk of the shuffled values (within the two central quartiles). For 2nd PC that proportion was only 6 of 14. Thus the experimental values for the loadings for the 1st and 2nd PCs are very unlikely to have arisen by change—and thus support the notion that the rehabilitative trends we detect using PCA are likely valid.

Similarly, the percentage of variance explained by the 1st and 2nd PCs of the experimental conventional and robot-assisted therapies are unlikely to have arisen by chance. We say this because a similar permutation tests shown in Additional file 1 shows that the actual values of variance explained found for the conventional and robot-assisted groups is not contained within the bulk of the shuffled values. Moreover, the fact that the variance explained by the 1st PC of the actual data was higher than for the shuffled values, and vice versa for the 2nd PC, indicates that the experimental data have more “structure” than the shuffled data. By more structure we mean a stronger departure from randomness because the percent of variance explained by the 1st and 2nd PCs are more dissimilar than in the shuffled data. These acceptable heuristic nonparametric interpretations strongly suggest the rehabilitative trends are not random, and differ between the robot-assisted and conventional therapies. Apropos the robustness of our results, an often-mentioned advantage of robotic therapy is that it can better standardize therapeutic delivery and dosage, which is harder to accomplish in multi-center, multi-therapist delivery of conventional therapy. However, the robustness of our results as tested by the random shuffling of patients into two groups (see Additional file 1), shows that any variability introduced by differences in conventional therapy across centers and therapist did not wash out the difference in rehabilitative trends between the two therapies.

Another more geometrically intuitive measure of the uniqueness of each rehabilitative trend is the direction of its PC vector in 7-dimensional space. As show in Fig. 1, each rehabilitative trend is a PC vector in the 7-dimensional space of changes in outcomes. The similarity between any two vectors in that space is found by the dot product of their unit vectors. This produces a value between 1 (parallel or identical) and 0 (perpendicular or most dissimilar), which corresponds to included angles of 0° and 90°, respectively. As shown in the Additional file 1, for each of the random shuffling of patients into groups A or B, we dotted the 1st and 2nd PCs with the 1st and 2nd PCs from the actual experimental assignment of patients. We find the experimental 1st and 2nd PCs are on average at least 40° away from their respective PCs in the shuffled data. Therefore, the PC vectors from the real data are not similar to the vectors arising from a random grouping of patients.

## Discussion

We interpret these results as demonstrating that robot-assisted and conventional therapies each produce different rehabilitative trends. To begin with, in robot-assisted therapy we see that improvements of motor function in the clinical environment (FMA, the a priori primary outcome of this study) can occur without concomitant improvements of function in the natural environment (i.e., MAL and SIS, the 1st PC of robot-assisted therapy). Conversely, functional improvements in the natural environment (MAL) can occur without improvements in motor function in the clinical environment (2nd PC of both therapies). Even if one is tempted to infer that the rank order of the 1st and 2nd trends is simply reversed across therapies (i.e., we see all positive loadings in the 1st PC of the conventional, and the 2nd PC of the robot-assisted therapies), we still see sign differences in the loadings across PCs, further indicating they are different. While future work is necessary to establish how robust these trends remain in the long-term, these results already allow us to make our two main claims: the numerical robustness of different rehabilitative trends in the short-term, and that that using a single primary outcome as the sole criterion for clinical relevance fails—at the very least—to consider the multi-dimensional nature of short-term recovery.

The limitations of PCA (see below) and the heterogeneity of conventional therapy in this study are a limitation that, nevertheless, do not challenge our result that robot-assisted and conventional therapies each produce different rehabilitative trends. In contrast to conventional therapy, robotic therapy can—in principle—better standardize therapeutic delivery across patients and centres. This is harder to accomplish in multi-center, multi-therapist delivery of conventional therapy. However, the robustness of our results as tested by the random shuffling of patients into two groups (see Additional file 1), shows that the unavoidable variability introduced by, say, measurement error, inter-subject differences, or differences in conventional therapy across centres did not wash out the difference in rehabilitative trends between the conventional and robot-assisted therapies.

Before discussing the results in detail, there are several methodological issues to consider given that interpreting PCA requires a certain degree of analytical nuance. As with any dimensionality-reduction technique [18], one must be careful not to over-interpret the results of PCA. We emphasize that our interpretation of the PCA results—and their robustness to data shuffling—pertains only to the demonstration that robot-assisted and conventional therapies each produce different rehabilitative trends. Going beyond this to evaluate the number of PCs to consider, and to interpret their individual loadings, requires care. As to the first issue, PCs are defined and listed in order of descending importance (i.e., percent of variance explained); and determining how many PCs to consider depends on the nature of the question. If one is interested in the number of PCs necessary to provide an equivalent—but lower dimensional or more compact—representation of the data, researchers in the field of motor control usually use as many PCs as necessary to explain 60 to 80 % of the variance in the data [18, 20]. However, our goal here is simply to demonstrate that the dominant trends (i.e., PCs that suffice to account for 50 % of the variance) are distinct across the two rehabilitation groups. Given that we are not making an argument about the amount of dimensionality reduction, the first two PCs suffice to establish differences in the dominant trends. The 3rd to 7th PCs each naturally explains an additional and decreasing amount of variance in the results, ranging from 16 to 5 %. But interpreting them becomes increasingly unclear, and they are not necessary or useful to establish differences in the dominant rehabilitative trends. Although we refrain from discussing them in detail, the supplemental material further show that the five remaining PCs continue to show differences between therapies—which reinforces our main finding.

As to the second issue, interpreting the loadings of each PC must be done carefully. That is, the PCs are orthogonal basis vectors that are equivalent to the semi-principal axes of an ellipsoid centered on the centroid of the data cloud (Fig. 1). Therefore, by the linear nature of PCA, PCs are orthogonal to each other and their loadings contain positive and negative values. This is a well-known drawback of PCA when applied to data where negative values or correlations do not make much sense. These cases include studies of neuron firing rates or muscle activations that are presumably always positive [21, 22], or as in this study where positive improvements with therapy are presumably expected. In this our first multi-dimensional comparison of rehabilitation strategies, we opted to use PCA (rather than other techniques like nonnegative matrix factorization [21]) because we did not want to make rigid positivity assumptions; and because PCA is a straightforward linear technique well-suited to a first attempt to ask whether robot-assisted and conventional therapies each produce different rehabilitative trends. Our main result does not depend on the details of the loadings or the orthogonality of the PCs. Thus our interpretation of the loadings is preliminary and serves mostly to guide future work; and negative loadings do not necessarily mean that some outcomes decrease as others increase—but rather that their 7-dimensional covariance is best described by a 7-dimensional ellipsoid with those semi-principal axes. Future studies could and should explore these results with other linear and nonlinear techniques.

What can be the reasons for—and clinical consequences of—these different rehabilitative trends? These trends could be influenced, at least in part, by differences in expectations on the part of the patients and therapists (including enthusiasm for novel devices and therapies, or higher affinity for human interaction), and by how these different expectations translate from activities in the clinical environment to activities in their natural environment. Additional work is needed to determine how clinically- and personally-significant these differences in trends are in the short- and long-terms, and how these multidimensional changes inform the selection, design, implementation and evaluation of each therapy.

We propose that these trends are in fact the result of the nature—and intended and unintended consequences—of each treatment protocol. Therapy sessions necessarily involve prioritization of activities due to considerations of rehabilitation philosophy and goals, time, cost, cognitive state, fatigue, etc. Of great interest is the fact that both therapies produce trends that include negative correlations among outcomes, which suggest the possibility of trade-offs in recovery across them. For example, conventional therapy includes strengthening exercises where the therapists resist the arm and encourages finger function. This may explain the high and medium correlation of changes in mean strength and grip strength to changes in clinical tests with (WMFTf and FMA in their 1st PC). In contrast, the robot-assisted therapy encouraged functional involvement of the limb in every day tasks, but did not emphasize limb strength. As commented recently [23], the antigravity support to encourage performance of simulated activities of daily living in the robot-assisted therapy (a personally stimulating goal) may have prevented strength increase in the paretic arm muscles (best promoted by rote strengthening exercises). This may explain the negative correlations between strength and clinical tests (FMA, WMFTf and WMFTt in their 1st PC). The trade-offs of each therapy may not only be responsible for differences in trends, but may also lead to different implicit and explicit expectations on the part of the therapist and patient. Likewise, the continued personal contact with an empathic clinician during conventional therapy may explain the consistently positive correlation of subjective quality of life outcomes (the two questionnaires MAL and SIS) with motor function (FMA in both the 1st and 2nd PCs of conventional therapy). In contrast, the robot-assisted therapy displays a potentially more objective, and less optimistic, correlation among motor function (FMA) and transfer to the natural environment (MAL and SIS). Such a dichotomy has been reported previously [24, 25]. Even so, the transfer of skills to daily life remains unclear because even within each of the therapies MAL and SIS are not consistently correlated with each other. Recent work shows that without the ‘transfer package,’ even the benefits of intense practice does not persist and transfer well to the natural environment [26].

Interestingly, changes in the primary outcome (FMA score) were the dominant effect only in one case: the 6th PC of patients receiving conventional therapy that explained only 8.51 % of the therapeutic effect (see Additional file 1). Changes in the Wolf Motor Function Test were often dominant in both robot-assisted and conventional therapies (i.e., one of its elements was the dominant factor in the 1st PC of both therapies). Therefore, one interpretation is that changes in Wolf Motor Function Test could be a more sensitive outcome than FMA, as has been proposed in [27, 28], at least in the mild to moderately impaired patients. Alternatively, it may also mean that the gains in Wolf Motor Function Test were more variable than the gains in other scores—and thus not necessarily a more sensitive outcome.

These results call for the development and evaluation of novel RCTs that leverage treatment protocols to exploit the different mechanisms of each rehabilitative trend. In spite of their strong technological and physiological foundations, previous rehabilitation RCTs emphasizing a single outcome implicitly assume a relatively narrow perspective to physical activity and its restoration. This is perhaps necessary to make them statistically rigorous and practical given the available resources, the robot-assisted implementation (e.g., automated repeated task practice), or the specific training and limitations of therapists. Thus there are often good reasons to select and declare a single primary outcome a priori (e.g., definition of sample size, comparison of studies) and this is even reflected in the *“Statistical Principles for Clinical Trials”* [29]. In each case, therapeutic choices and RCT designs (including the choice of the primary outcome measure) are necessarily the embodiment of a particular therapeutic philosophy, value system, and approach given the resources and skills available. The specific origins and perspectives of each therapy define the therapeutic focus in practice, but therapy must necessarily (explicitly and implicitly) affect multiple dimensions of body structure and function, activity and participation as per the ICF, Table 2. Our goal is not to replace current RCT philosophy, but rather to complement it using the additional information available in the multidimensional nature of function and recovery. This view complements the choice of a primary outcome measure (which remains a point of contention when comparing RCTs) by emphasizing that there is additional information in the multidimensional nature of function and recovery.

It is encouraging, therefore, that in some fields RCTs advocate and report the use of multiple patient-centered outcomes [30, 31]; and promising that we were able to identify different rehabilitative trends. This agrees with others who suggest that recovery may depend on processes that affect multiple interrelated abilities simultaneously, occurring at both global and task-specific levels [13, 32]. Therefore, our identification of distinct rehabilitative trends is likely not an isolated occurrence limited to the therapies we compared in this study. Rather, we propose that the choices inherent to any therapeutic approach will naturally produce a particular rehabilitative trend.

How should we leverage these rehabilitative trends to improve therapeutic outcomes? A single primary outcome measure has the clear advantage of lending itself to uni-dimensional power analysis of clinically meaningful changes. In fact, the dramatic drop in statistical power inherent to multi-dimensional analyses may be one of the forces driving the community’s preference for a single primary outcome. A single primary outcome measure may be the only statistically feasible means to design RCTs with a realistic number of subjects and cost. From this perspective, PCA appears to be most useful for retrospective or exploratory analyses, as it is less clear how it can be used at the outset during the design of an RCT. But this multidimensional perspective, even if retrospective and at odds with today’s univariate statistical formalism, can be made useful in subsequent studies or RCTs by guiding the programming of the robotic therapy, modifying the emphasis in the traditional therapy, and evaluating the relevance of the results to goals of the ICF’s. For example, our results have already emphasized the need to modify the robotic protocol to promote finger strength, and provided valuable information and motivation to compare and contrast the Fugl-Meyer and Wolf Motor Function tests in the context of other outcome measures. It also confronts us to understand the relationship between the worthwhile goals of the ICF, vs. the real-world limitations of individual outcome measures as reported recently [33, 34]. Moreover, this multidimensional approach to rehabilitative trends enables and complements the development and testing of multi-variable models of plasticity and motor learning in the context of the emerging field of computational neurorehabilitation [24].

While this work addresses a relevant problem in rehabilitation trials by using a novel retrospective analysis of all outcome measures, it is important to highlight its potential clinical utility going forward. Given that PCA is such a common and accessible analysis tool, we propose that investigators could revisit their databases of outcomes (which rarely include solely the primary outcome). A retrospective analysis as presented here may allow the extraction of additional information from in the multidimensional response to therapeutic intervention and functional recovery. That is, PCA should be used retrospectively to understand how the primary and secondary outcomes interact, and to set up future trials and mechanistic questions about how therapeutic interventions—robotic or not—impact outcomes. Perhaps especially in negative trials to understand why there were no differences in the primary outcome. In addition, future directions could include finding predictors of rehabilitative trends based on the initial presentation of the patient along one or multiple outcomes. Similarly, whether and how such presentation affects the nature of the rehabilitative trends is a question that is enabled by this approach. However, the scope of this first paper is limited to presenting the existence of such distinct multi-dimensional rehabilitative trends.

The concept of rehabilitative trends has important implications to the basic science and implementation of effective neuro-rehabilitative therapies. On the one hand, it enables the study of physiological and social psychological mechanisms that produce such interactions among outcomes. On the other, it encourages the application of this broader perspective to align the trade-offs and strengths of both robot-assisted and conventional therapies with the specific rehabilitative trends to meet the specific goals of the patient. Future RCTs should focus on what these distinct trends really mean and how one could use that information to shape the specific therapy for each individual patient.

## Conclusions

Our results demonstrate that robot-assisted and conventional therapies each produce different rehabilitative trends of recovery across the clinical, functional, and quality of life domains. A rehabilitative trend is defined as correlations among changes in outcomes with each therapy. These findings challenge the traditional emphasis of RCTs on using a single primary outcome measure to quantify and compare rehabilitative responses that are naturally multidimensional. This alternative approach to, and interpretation of, the results of RCTs may will lead to more effective therapies targeted for the multidimensional mechanisms of recovery.

## Additional file

Additional file 1: This supplemental material assess the robustness of the principal components analysisresults in the main text. (DOCX 889 kb)

## Abbreviations

ARMin: An exoskeleton robot that allows task-specific training in three dimensions with assistance as needed control
FMA: Fugl-Meyer Assessment
ICF: International Classification of Functioning, Disability and Health
MAL: Motor Activity Log. A structured interview with the patient about quality of movement of the affected arm in the natural, home, and community environment
PC: Principal component
PCA: Principal components analysis
PCs: Principal components
RCT: Randomized clinical trial
RCTs: Randomized clinical trials
SIS: Stroke Impact Scale. A combination of the four domains of strength, hand function, mobility, and activities of daily living of the complete SIS questionnaire
WMFTf: Wolf Motor Function Test function-domain. A q*ualitative* measure of motor performance of the affected arm in the clinical environment
WMFTt: Wolf Motor Function Test time-domain. A *quantitative* measure of performance of the affected arm in the clinical environment

## Footnotes

1 There are seven PCs because PCA is based on the covariance matrix among the seven outcomes, which has seven rows and columns.
