# Erratum to: Robot-assisted and conventional therapies produce distinct rehabilitative trends in stroke survivors

**DOI:** 10.1186/s12984-016-0210-1

**Published:** 2016-12-06

**Authors:** Francisco J. Valero-Cuevas, Verena Klamroth-Marganska, Carolee J. Winstein, Robert Riener

**Affiliations:** 1 Department of Biomedical Engineering, University of Southern California, 3710 McClintock Ave, RTH 404, Los Angeles, CA 90089-2905 USA; 2 Division of Biokinesiology and Physical Therapy, University of Southern California, Los Angeles, CA USA; 3 ETH Zurich and University of Zurich, Zurich, Switzerland

## Erratum

The original article [1] contains an error in the **Conclusions** section.

The original article states the following:

“This alternative approach to, and interpretation of, the results of RCTs will lead to more effective therapies targeted for the multidimensional mechanisms of recovery.”

This should instead say:

“This alternative approach to, and interpretation of, the results of RCTs **may** lead to more effective therapies targeted for the multidimensional mechanisms of recovery.”
