# Supplementary material for: Robot-assisted and conventional therapies produce distinct rehabilitative trends in stroke survivors
Source: J Neuroeng Rehabil. 2016 Oct 11;13:92. doi: 10.1186/s12984-016-0199-5 (PMC5057463; doi:10.1186/s12984-016-0199-5)
Supplement: Additional file 1: — This supplemental material assess the robustness of the principal components analysisresults in the main text. (DOCX 889 kb) [file 12984_2016_199_MOESM1_ESM.docx]

Supplementary Appendix for:

Robot-assisted and Conventional Therapies Produce
Distinct Rehabilitative Trends in Stroke Survivors

**Francisco J. Valero-Cuevas1,2, Verena Klamroth-Marganska3,
Carolee J Winstein2, and Robert Riener3**

1Department of Biomedical Engineering, 2Division of Biokinesiology and Physical Therapy, University of Southern California, Los Angeles, CA, USA
3Swiss Federal Institute of Technology (ETH), Zurich, Switzerland

We used principal components analysis (PCA) to obtain the rehabilitative trends between the start and end of eight weeks of therapy. Using the change in each of the outcomes between the start and end of each therapy (Table 1 of the manuscript), PCA finds the best linear fit to the data—shown in Figure 1 of the manuscript using a schematic in three dimensions. The visualization of a hyperplane embedded in seven dimensions is not possible to show graphically, but the intuition obtained in the three dimensional schematic example carries over to higher dimensions. The PCs are the vectors of correlations that describe the seven dimensional changes in outcomes that best explain the results of the randomized clinical trial. Each of the seven principal components (PCs) is a column vector as shown in Tables 1 & 2 of the manuscript.

Each PC represents a rehabilitative trend as it quantifies how the correlations among changes in outcomes explain their total change (i.e., variance) with therapy. PCs are rank-ordered, with the first explaining the most variance, and the seventh the least. The entries of each PC, called loadings, specify the details of each rehabilitative trend. Tables 1 & 2 show the loadings and variance explained for all seven PCs. The manuscript only discusses the first two PCs in detail because they suffice to explain ~50% of the variance. The 3^rd^ to 7^th^ PCs each explains a decreasing amount of variance in the results, ranging from 16% to 5%. Thus interpreting them becomes increasingly unclear. Therefore, we refrain from doing so and only point out that the five remaining PCs continue to show differences between therapies—which is our main finding.

Our results further show that the 1^st^ and 2^nd^ rehabilitative trends are robust because they are unlikely to appear by chance. We did so by using a permutation test [^1^](#_ENREF_1) that randomly and repeatedly shuffled patients into two groups, A and B, of size equivalent to the conventional and robot-assisted therapies. That is, we performed 100 iterations of our PCA analysis where, for each shuffling, we assigned patients to one of two groups at random. We found a low probability of replicating the actual 1^st^ and 2^nd^ PCs for each therapy. As show in Figure S1, the percentage of variance explained by the 1^st^ and 2^nd^ PC of the conventional and robot-assisted therapies has a low probability of appearing by chance. The variance explained (asterisks) of the actual therapies lie outside of the central quartiles of the shuffled groups A and B, and thus occur <75% of the time in the shuffled data. Moreover, there is a stronger tendency towards a non-uniform structure in the experimental groups than in the shuffled groups. This is evidenced by the fact that the variance explained by the actual 1^st^ PCs is higher than in the shuffled groups, and vice versa for the 2^nd^ PCs. By comparison, therapies with similar effects would lead to a more uniform distributions of variance explained across all PCs. That is, the distribution of variance in the experimental groups exhibits more—not less—structure that is expected to occur by chance.

**
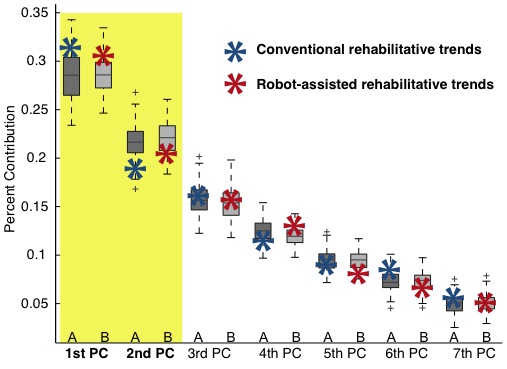
**

**Figure S1.** Box plots of variance explained by all seven PCs after patients are randomly shuffled 100 times in to groups A and B. Note the actual experimental values of variance explained by the 1^st^ and 2^nd^ PCs (asterisks) do not lie within the two central quartiles, and thus have a low probability of appearing by chance. The 3rd to 7th PCs are included for completeness, but we refrain from interpreting them as they each explain increasingly less variance.

In addition, data shuffling allowed us to test the robustness of the details of each rehabilitative trend, which is given by the loadings of each PC as shown in Figure 1 of the Manuscript. Figure S2 now shows that the loadings of the 1^st^ and 2^nd^ PCs (i.e., the two main rehabilitative trends) show distinct departures from the loadings seen in the 100 shufflings into groups A and B. Shuffling data repeatedly would naturally tend to produce loadings that are similar across both groups A and B, which can be seen by the clear overlap of box plots in Figure S2. However, the actual rehabilitative trends we report show clear departures from this uniformity.

Lastly, a more quantitative and intuitive measure of differences between seven-dimensional rehabilitative trends in the real data compared to those in the shuffled groups is the angle between their PC vectors. Each rehabilitative trend is a PC vector in 7-dimensional space, and the similarity between two vectors is found by the dot product of their unit vectors. This produces a value between 1 (parallel or identical) and 0 (perpendicular or most dissimilar), which intuitively corresponds to included angles of 0° and 90°, respectively. For each of the random assignments of patients into groups A or B, we dotted their 1^st^ and 2^nd^ PCs with the actual 1^st^ and 2^nd^ PCs from the real assignment of patients. Figure S3 shows that the actual experimental 1^st^ and 2^nd^ PCs are always far from having a zero angle with their respective PCs in the shuffled data (in general >40° except for the 1^st^ PC for traditional therapy). Therefore, the PC vectors from the real data are not similar to the vectors arising from a random grouping of patients. Taken together, these analyses strongly support our interpretation that the experimental rehabilitative trends we report are robust, and that each of the therapies produces distinct rehabilitative trends.

**Figure S2.** The loadings of the two main rehabilitative trends of the conventional and robot-assisted therapies show distinct departures from those of a random shuffling of patients into groups A and B. Note that few of the actual experimental loading values lie close to the median or in the two central quartiles of the shuffled data.

**Figure S3.** The included angle (left scale) between the actual experimental PCs and each of their respective PC in the shuffled data quantify their degree of similarity (right scale). We find that the main rehabilitative trends, the 1^st^ and 2^nd^ PCs, of the conventional and robot-assisted therapies show large angles (a median of ~50% similarity for three out of four cases, ~80% similarity for the 1^st^ PC of conventional therapy) from those of a random shuffling of patients into groups A and B.

**References**

1. Good PI. Permutation, parametric and bootstrap tests of hypotheses. 3rd ed. New York: Springer; 2005.
